# A neurocognitive model of perceptual decision‐making on emotional signals

**DOI:** 10.1002/hbm.24893

**Published:** 2019-12-23

**Authors:** Mihai Dricu, Sascha Frühholz

**Affiliations:** ^1^ Department of Psychology University of Bern Bern Switzerland; ^2^ Cognitive and Affective Neuroscience Unit, Department of Psychology University of Zurich Zurich Switzerland; ^3^ Neuroscience Center Zurich (ZNZ) University of Zurich and ETH Zurich Zurich Switzerland; ^4^ Center for Integrative Human Physiology (ZIHP) University of Zurich Zurich Switzerland

**Keywords:** amygdala, decision‐making, emotion, fMRI, neural network, perception

## Abstract

Humans make various kinds of decisions about which emotions they perceive from others. Although it might seem like a split‐second phenomenon, deliberating over which emotions we perceive unfolds across several stages of decisional processing. Neurocognitive models of general perception postulate that our brain first extracts sensory information about the world then integrates these data into a percept and lastly interprets it. The aim of the present study was to build an evidence‐based neurocognitive model of perceptual decision‐making on others' emotions. We conducted a series of meta‐analyses of neuroimaging data spanning 30 years on the explicit evaluations of others' emotional expressions. We find that emotion perception is rather an umbrella term for various perception paradigms, each with distinct neural structures that underline task‐related cognitive demands. Furthermore, the left amygdala was responsive across all classes of decisional paradigms, regardless of task‐related demands. Based on these observations, we propose a neurocognitive model that outlines the information flow in the brain needed for a successful evaluation of and decisions on other individuals' emotions.

**Highlights:**

- Emotion classification involves heterogeneous perception and decision‐making tasks
- Decision‐making processes on emotions rarely covered by existing emotions theories
- We propose an evidence‐based neuro‐cognitive model of decision‐making on emotions
- Bilateral brain processes for nonverbal decisions, left brain processes for verbal decisions
- Left amygdala involved in any kind of decision on emotions

## 1 INTRODUCTION

**Figure 1:**
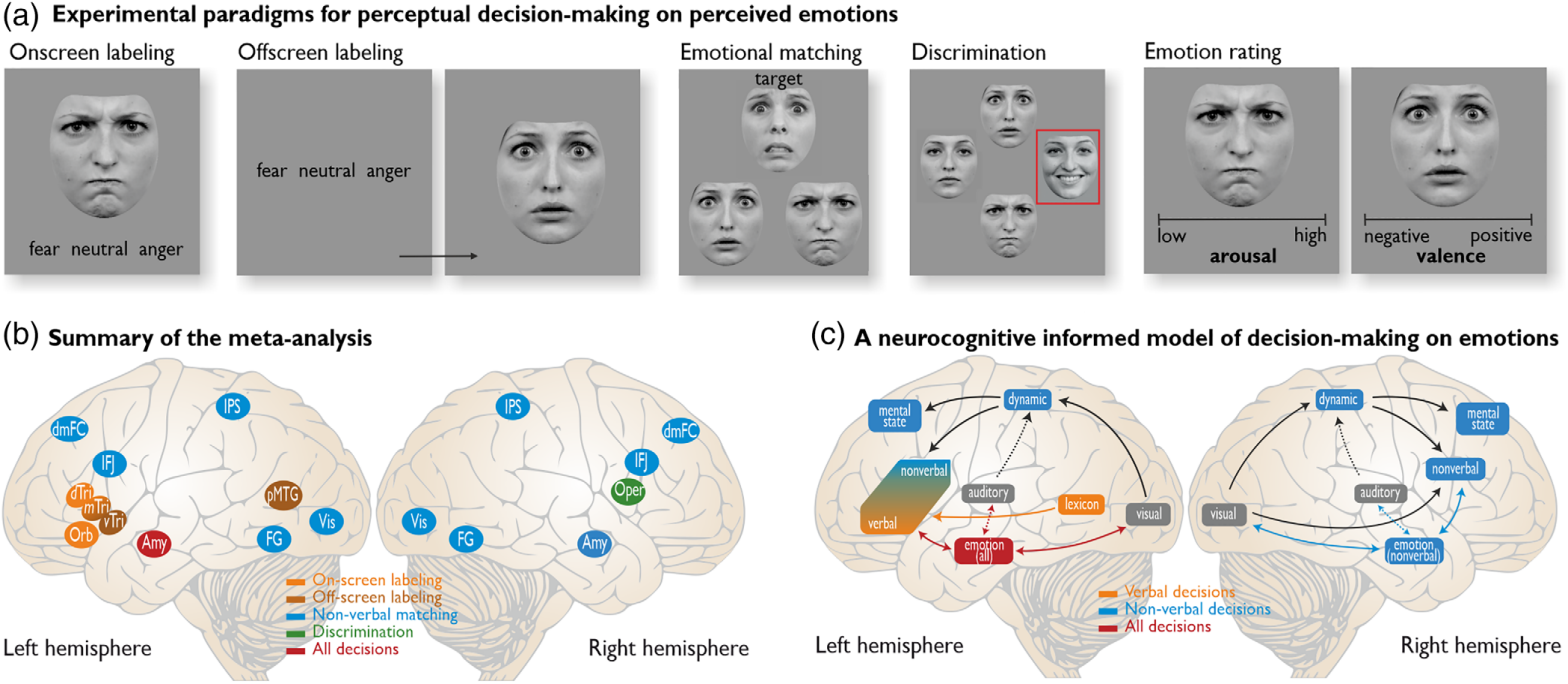
(a) Paradigms of perceptual decision‐making on others' emotional expressions. On‐screen labeling (top left) consists of matching the perceived emotional expression to a verbal label that is simultaneously displayed on‐screen. In off‐screen emotion labeling (top middle), participants are asked to keep a mental trace of the possible verbal labels throughout the experiment and match the perceived expression to the correct label. The emotion matching task (top right) consists of a triad of facial expressions, in which participants must match the expression of the target face to the expression of one of two simultaneously presented faces. Emotion rating asks participants to rate the level of arousal (bottom left) or valence (bottom middle) of the emotional expression. The bottom right corner depicts a variant of emotion discrimination (“same or different” task), in which participants must determine whether two target stimuli portray identical or different emotional expressions. For ease of illustration, all stimuli depicted here consist of facial expressions of emotions. However, except for emotion matching, which consists exclusively of facial expressions, the stimuli included in our meta‐analyses consisted of facial expressions, vocal prosody, and body postures. (b) Summary of the findings on perceptual decision‐making on emotions, with a special focus on the unique regions revealed by the contrast analyses. (c) A neurocognitive model of decision‐making on emotions based on general principles of perceptual processing connecting sensory regions (visual, auditory), association areas (lexicon, dynamic), and limbic areas (emotion) with higher‐cognitive areas in the frontal cortex (mental state, verbal, nonverbal). Amy, amygdala; dMFC, dorsomedial frontal cortex; dTri, dorsal pars triangularis; FG, fusiform gyrus; IFJ, inferior frontal junction; IPS, intraparietal sulcus; mTri, mid pars triangularis; Oper, pars opercularis; Orb, pars orbitalis; pMTG, posterior middle temporal gyrus; Vis, visual cortex; vTri, ventral pars triangularis

The process of perceiving and identifying emotions signaled by others is often a split‐second instance of emotion perception. However, this apparently rapid action is actually the outcome of multiple stages of decision‐making based on various levels of neural and cognitive processing. This decisional process might differ according to the specific requirements of certain contexts and situations. Some contexts, for example, require the perceiver to label the emotions they recognize in another to communicate them to others involved, thereby transposing a sensory percept into a verbal category (i.e., verbal labeling). Other contexts might require more basic types of recognition below the level of verbalizations, such as matching the emotions of two individuals (i.e., emotional matching) or deciding that one person shows an emotion different from others or from previous encounters (i.e., emotional discrimination). Finally, other contexts might only require rating the intensity of emotions regardless of the emotion perceived (i.e., emotional intensity rating). These different types of decisions on perceived emotions are assumed to imply different neurocognitive mechanisms (Figure 1).

During these various situational types of decision‐making on perceived emotions, the human neurocognitive system needs to extract sensory information from different sensory channels, such as facial and vocal expressions and body postures, integrate this data into a gestalt percept, and then interpret it (Belin, Fecteau, & Bedard, 2004; Bernstein & Yovel, 2015; de Gelder, De Borst, & Watson, 2015). The full process of extraction, integration, and interpretation of sensory information enables individuals to perform perceptual decisions about the most likely emotion expressed by other individuals (i.e., categorization of facial, vocal, or bodily features as expressing an emotional state, e.g., joy). This process of deliberation in which sensory information is used to decode and evaluate the external world is called perceptual decision‐making (Hauser & Salinas, 2014; Heekeren, Marrett, Bandettini, & Ungerleider, 2004; Mulder, van Maanen, & Forstmann, 2014; Schall, 2001). In contrast to other forms of decision‐making, perceptual choices emphasize the role of sensory information in reaching a decision and in directing reactive behavior (Summerfield & De Lange, 2014; Wiech et al., 2014).

There are currently no formal detailed neurobiological, neurocognitive, or psychological models of how humans perceive emotions in individuals. The present work aims to fill this gap of knowledge. Much has been said by biological, neurocognitive, and psychological theories concerning emotion elicitation and emotion expression, but few of these theories directly address the process of perceiving emotions in other individuals (e.g., Coppin & Sander, 2013; Faucher, 2013; Nesse, 2014). For example, affect program theories postulate that individuals from different cultures and even species are born with the same capabilities of expressing emotions through sets of motor responses, such as facial and vocal expressions and body postures (Ekman et al., 1987). In return, this makes emotion perception and inference possible with a high level of certainty without the use of any other information (Ekman, 1992; Ekman et al., 1987; Panksepp, 2000; Scherer, Clark‐Polner, & Mortillaro, 2011). Strong appraisal theories, on the other hand, postulate that we are capable of inferring the emotions of others by reverse‐engineering the individual appraisal patterns associated with each perceived emotional expression (Scherer & Ellgring, 2007; Scherer, Mortillaro, & Mehu, 2013). For example, upon encountering someone's facial expression of wide eyes and open mouth (i.e., common expressions for both surprise‐inducing and fear‐inducing stimuli; Scherer & Ellgring, 2007), we can deduce that the individual appraises the stimulus as novel and unexpected but not as threatening because no fear‐related expressions follow, such as backing up and moving away. Nevertheless, the exact mechanism of inferring the emotional experiences in others remains vaguely formulated in both appraisal theories and affect program theories (Ekman & Cordaro, 2011; Scherer, Banse, & Wallbott, 2001; Scherer & Ellgring, 2007), though it has been proposed that the act of emotion inference is separate from the act of perception (Scherer & Ellgring, 2007; Scherer et al., 2013).

Perhaps the one account that has come closest to providing a mechanistic account of emotion perception consists of constructivism theories, which argue that others' emotions can be accurately inferred from a combination of motor expression perception, context processing and conceptual knowledge about the relationships between emotions, desires, and beliefs (Barrett, 2006; Barrett & Kensinger, 2010; Russell, 2005, 2009). The perception of motor expressions can inform the emotion inference process not because motor expressions reflect emotions per se (i.e., there is no one‐on‐one mapping) but because the perceiver has learned through experience to associate certain motor expressions with certain emotional experiences via a bootstrapping process (Barrett, Mesquita, & Gendron, 2011; Lindquist, 2013) or even a process of elimination (DiGirolamo & Russell, 2017; Nelson & Russell, 2016). For example, individuals may have come to learn that frown eyebrows and pouted lips are often associated with a limited set of mental states (e.g., anger and sudden euphoric joy). Here, the perception of context is crucial in determining which of the possible mental states from the set is the most likely felt emotion (Barrett & Kensinger, 2010; Barrett et al., 2011; Sommer, Dohnel, Meinhardt, & Hajak, 2008). Finally, constructivism theories argue that conceptual knowledge in the form of folk theories about emotions and mental states can help refine the inference process (Barrett, 2006; Lindquist, 2013; Ochsner et al., 2009; Zaki, 2013) and that language processes play a crucial role in the categorical perception of emotional expressions (Barrett, Lindquist, & Gendron, 2007; Lindquist, Barrett, Bliss‐Moreau, & Russell, 2006; Lindquist & Gendron, 2013). However, this evidence is not uncontroversial (Deonna & Scherer, 2010; Panksepp, 2007; Sauter, 2018; Sauter, LeGuen, & Haun, 2011).

In summary, the majority of psychological and neurocognitive theories only go as far as describing how an individual experiences and manifests an emotion, which represents only the input for the perceptual decision‐making process on others' emotions (Scherer et al., 2013). One possible reason for this discrepancy in theory coverage is the overwhelming evidence that the emotional expressions of others are perceived in a categorical manner (Fugate, 2013; Jaywant & Pell, 2012), which is compatible with multiple theories of emotion (e.g., Scherer et al., 2011), hence providing little incentive for further scrutiny. The term categorical perception describes the subjective experience in which a perceived dimension jumps abruptly from one category to another at a certain point along a continuum, instead of changing gradually (Liberman, Harris, Hoffman, & Griffith, 1957). For example, faces or voices in a morphing sequence between two prototypical emotions are perceived as either one or the other but not as something in between (Cheal & Rutherford, 2015; Etcoff & Magee, 1992; Fujimura, Matsuda, Katahira, Okada, & Okanoya, 2012; Jaywant & Pell, 2012; Korolkova, 2014; Laukka, 2005). The robust phenomenon of categorical perception of emotions has been replicated with various response formats and analysis methods (Bimler & Kirkland, 2001; Campanella, Quinet, Bruyer, Crommelinck, & Guerit, 2002; Cheal & Rutherford, 2010, 2015; Dailey, Cottrell, Padgett, & Adolphs, 2002; Fujimura et al., 2012; Kotsoni, de Haan, & Johnson, 2001; Sauter et al., 2011). Categorical and continuous perception often co‐occur when processing the emotions of others: the former allows for a gestalt perception of a single emotion, while the latter enables to us to perceive subtle variances within an emotional construct (Fujimura et al., 2012). However, categorical perception appears to dominate the way we process and attribute emotions in others (Fugate, 2013) and the reason for this could be to achieve cognitive efficiency by parsing out information into meaningful, but limited, pieces of information (Goldstone & Hendrickson, 2010; Harnad, 1987; Schusterman, Reichmuth, & Kastak, 2000). Indeed, Etcoff and Magee (1992) argued that, if the perceiver were to quickly detect the sender's mental state, a blend of emotions would be difficult to interpret meaningfully. Instead, relying on the dominant emotion in the signal would be more likely to give an accurate prediction about the sender's mental state and, by proxy, about the environment.

The highly debated question in the emotion perception field is not *whether* emotions are perceived categorically, but rather to what extent is the phenomenon of categorical perception *more perceptual* or *more conceptual* (Fugate, 2013). In other words, do we subjectively perceive others' emotional expressions as discrete entities because emotions per se are discrete categories or is it because we constructively create the perceived emotion so effortlessly out of multiple sources of information (e.g., context, knowledge about the target) that our brains are “tricked” into seeing distinct categories, akin optical illusions? As with many psychological phenomena, a middle ground has been proposed: there is a seesaw relationship between the innate tendency for categorical perception and the context in which the emotional expression occurs (Fugate, 2013; Hess & Hareli, 2017). As the emotional signal increases in noise and becomes ambiguous (e.g., tendency of the perceived agent to mask the emotion), so does the influence of context and language increases in deducing the emotional state. Conversely, the richer the emotional expression is in situational information (e.g., a boisterous laugh), the lower the need to rely on context to infer the respective emotional state (Hareli, Elkabetz, & Hess, 2019).

Lesion and neuroimaging studies are principally equipped with informing models of perceptual decision‐making because they aim to reveal underlying brain structures and disclose associated mental processes, an impossible feat for behavioral or physiological experiments (Aue, Lavelle, & Cacioppo, 2009). However, as with biological and psychological models of emotions, current neurocognitive models suffer from a similarly unbalanced focus on the input of perceptual decision‐making on emotions, namely the type of sensory information that is extracted by the visual and auditory cortices (Belin et al., 2004; Concina, Renna, Grosso, & Sacchetti, 2019; Frühholz, Trost, & Kotz, 2016; Haxby, Hoffman, & Gobbini, 2000; Rauschecker, 2017; Sedda & Scarpina, 2012) and how this information is integrated into a percept (Bernstein & Yovel, 2015; Brück, Kreifelts, & Wildgruber, 2011; Heekeren, Marrett, & Ungerleider, 2008; Schirmer & Adolphs, 2017; Schirmer & Kotz, 2006). So far, less focus has been placed on how higher cognitive functions (e.g., language processes, accessing semantic knowledge) contribute to the formation of a holistic percept and its interpretation within various contexts.

Perceptual decision‐making on emotions is naturally part of a general perception system, for which neurocognitive models do exist. However, simple extrapolation from these models to the realm of emotional expressions is not warranted, as the latter constitute a special class of signals while the former were built from generic stimuli (Sander, Grafman, & Zalla, 2003; Scherer, 2009; Van Kleef, 2010). Unlike perceiving colors and pure tones, for instance, evaluating emotional stimuli engages a broader range of cognitive processes and potentially higher degrees of perceptual and inferential freedom. Nevertheless, we expect that perceptual decision‐making on emotional expressions adheres to the same three principles of general perception. First, sensory information is extracted by visual and auditory primary and associative regions (Hauser & Salinas, 2014). Within these regions, there are further areas specialized in processing human faces (Bernstein & Yovel, 2015; Haxby et al., 2000), body postures (de Gelder et al., 2015; Peelen & Downing, 2005), and human voices (Belin et al., 2004; Ceravolo, Fruhholz, & Grandjean, 2016; Frühholz & Belin, 2018; Pernet et al., 2015). Second, this sensory information is passed along two anatomically segregated and functionally specialized processing streams, dubbed the ventral stream and the dorsal stream (Goodale & Milner, 1992; Goodale & Westwood, 2004; O'Reilly, 2010; Rauschecker, 2012, 2013). The ventral pathway, connecting primary sensory cortices with temporal and prefrontal regions, is functionally conceptualized as the “what” stream, responsible for stimulus recognition and identification, and the mapping of sensory information onto conceptual representations (Goodale & Milner, 1992; Grill‐Spector & Weiner, 2014; Hebart & Hesselmann, 2012; Kravitz, Saleem, Baker, Ungerleider, & Mishkin, 2013). The dorsal pathway, connecting primary sensory areas with parietal and prefrontal regions, constitutes the “where/how” stream, responsible for processing space and motion, including locating objects in space, understanding others' movements and guiding our own actions toward objects in space (Arbib, 2017; Friederici, 2012; Goodale, Westwood, & Milner, 2004; Lega, Stephan, Zatorre, & Penhune, 2016; Murakami, Kell, Restle, Ugawa, & Ziemann, 2015). Finally, perception occurs when the incoming sensory information is made available to higher‐order brain regions and matched against a mental template. In the ventral stream, this mental template consists of semantic categorical representations (a prototype of a stimulus, e.g., how a face generally looks like) (Sedda & Scarpina, 2012; Summerfield & Koechlin, 2008; Summerfield et al., 2006; Takahashi, Ohki, & Kim, 2013). In the dorsal stream, the mental template consists of visuomotor and audiomotor sequences potentially stored in our procedural memory (e.g., how emotional expressions and emotional utterances evolve over time) (Goodale, Króliczak, & Westwood, 2005; Goodale & Milner, 1992; Rauschecker, 2011, 2012). Such templates allow us to perceive and discriminate other individuals' actions, including facial movements (Bernstein & Yovel, 2015) and speech (Rauschecker, 2012).

A later stage in the emotional perception process concerns emotional categorization or verbal labeling. By converting the set of sensory information into a percept that can be communicated, an individual is able to relate and describe the emotional status of another individual. Regarding this stage, a significant task‐dependent role is assigned to the frontal cortex in matching incoming sensory information to a mental template (Brück et al., 2011; Dricu & Frühholz, 2016; Frühholz & Grandjean, 2013b; Liakakis, Nickel, & Seitz, 2011; Sakagami & Pan, 2007; Schirmer & Kotz, 2006). While reviews and meta‐analyses exist on the multiple roles of the inferior frontal cortex (IFC) in perceiving a large class of stimuli (Dal Monte et al., 2014; Greenlee et al., 2007; Liakakis et al., 2011; Rahnev, Nee, Riddle, Larson, & D'Esposito, 2016), no such systematic reviews exist for perceiving emotional expressions, despite abundant empirical data (Dricu & Frühholz, 2016). Another consistent frontal brain structure recruited during the perceptual decisions on emotions is the dorsomedial frontal cortex (dmFC). This structure is predominantly involved in social cognition, such as forming impressions about others and inferring beliefs, desires, and intentions (Fruhholz, Trost, & Grandjean, 2016; Korb, Fruhholz, & Grandjean, 2015; J. P. Mitchell, Cloutier, Banaji, & Macrae, 2006; Schlaffke et al., 2015; Schurz, Radua, Aichhorn, Richlan, & Perner, 2014; Venkatraman, Rosati, Taren, & Huettel, 2009). The emotional expressions conveyed by others are inherently social stimuli and are processed differently from other classes of stimuli such as inanimate objects (Amodio & Frith, 2006). The involvement of the dmFC in this particular decisional process suggests that facial, bodily and vocal emotional expressions are not only proxies for mental states but also that perceivers spontaneously infer traits and mental states (i.e., beliefs, desire, intention) (Reisenzein, 2009), which are integrated in the emotional evaluation (Dricu & Frühholz, 2016; Uleman, Newman, & Moskowitz, 1996; Van Overwalle, 2009, 2011).

In addition to the frontal brain structures targeted by the ventral and dorsal processing stream, other brain structures that do not exclusively belong to either processing stream also contribute to perceptual decisions on emotions. One such structure is the amygdala, which works concomitantly with sensory cortices and higher‐order cortices to tag incoming sensory information with contextual relevance (Pannese, Grandjean, & Fruhholz, 2016; Phelps & LeDoux, 2005) and subsequently detect this relevance upon the next encounter with that stimulus or circumstance (Sander et al., 2003). For example, the procedure of fear conditioning instills an initially neutral stimulus with the capacity of inducing reactions and behaviors that are biologically relevant (e.g., freezing or fleeing) upon consistent association with an aversive unconditioned stimulus (Pape & Pare, 2010). Furthermore, the amygdala is likely to process diverse emotional expressions, such as facial expressions (Haxby, Hoffman, & Gobbini, 2002; O'Toole, Roark, & Abdi, 2002; Rossion, 2015; Sabatinelli et al., 2011) and vocal prosody features (Fruhholz, Klaas, Patel, & Grandjean, 2015; Frühholz et al., 2015, 2016; Pannese, Grandjean, & Frühholz, 2015, Pannese et al., 2016) as relevant social signals (Sander et al., 2003). The large variety of cortical and subcortical projections to and from the amygdala provide it with information about the properties of the stimulus as well as the ongoing goals and needs of the organism (J. L. Price, 2003). As such, the amygdala might serve as one of the interfaces between sensory cortices and higher‐order brain structures.

Altogether, perceptual decision‐making on emotions likely involves a large neural network of brain regions with complementary functional roles. The present meta‐analysis endeavored to systematically review and meta‐analytically analyze the neuroimaging literature to uncover our knowledge of this large neural network to date. Additionally, we also aimed to account for several shortcomings in the field of emotion perception. The first shortcoming concerns a lack of acknowledgment of the heterogeneity of perceptual tasks on emotional expressions. The second is the lack of interest on the differential involvement of distributed brain systems depending on the decisional requirements. There has been an implicit assumption that perception tasks do not differ qualitatively from one another (Elliott, Zahn, Deakin, & Anderson, 2011; Ong, Zaki, & Goodman, 2015; Schlegel, Boone, & Hall, 2017). Reviews and meta‐analyses frequently aggregate heterogeneous tasks of emotion perception, discarding any differences in task instructions (Fusar‐Poli, Placentino, Carletti, Landi, & Abbamonte, 2009; Müller, Höhner, & Eickhoff, 2018; Phan, Wager, Taylor, & Liberzon, 2002; Wager, Phan, Liberzon, & Taylor, 2003). Alternatively, some researchers use a specific paradigm of emotion perception and then extrapolate their findings across the entire phenomenon of emotion perception (e.g., (Adolphs, Damasio, Tranel, Cooper, & Damasio, 2000).

Notably, in a recent meta‐analysis, Müller et al. (2018) examined the influence of task requirements (i.e., explicit evaluation of facial emotional expression vs. focus on a nonemotional face feature) on the recruitment of brain regions during human imaging studies. However, their focus was solely on the visual face‐processing network and they did not differentiate between different types of explicit evaluation and decision tasks. To fill in these gaps, we review both the visual and auditory domain of others' emotional expression as part of the decisional process on emotions. Furthermore, we specifically argue that the differential requirements of various explicit emotion perception tasks including decisions on perceived emotions should be assessed. While neurobiologists and neuroscientists have long used sophisticated batteries of tests that tap into various facets of emotion perception in studies, for both healthy participants and patient samples (Boller & Grafman, 2000; Wilhelm, Hildebrandt, Manske, Schacht, & Sommer, 2014), there still seems a certain lack of acknowledgment concerning the heterogeneity of perceptual and decisional tasks on emotional expressions and their differential neural implications. We thus reviewed the neuroimaging literature of emotion perception spanning 30 years. Using the existing literature to inform us about the neurocognitive mechanisms behind perceptual decisions on others' emotions, we then built an evidence‐based neurocognitive model of emotion perception (Figure 1). Finally, we connected our neurocognitive model of emotion perception with biological, neuroscientific, and psychological theories of emotion and neurocognitive models of general perception.

## 2 MATERIALS AND METHODS

### 2.1 Selection of neuroimaging studies

**Figure 2:**
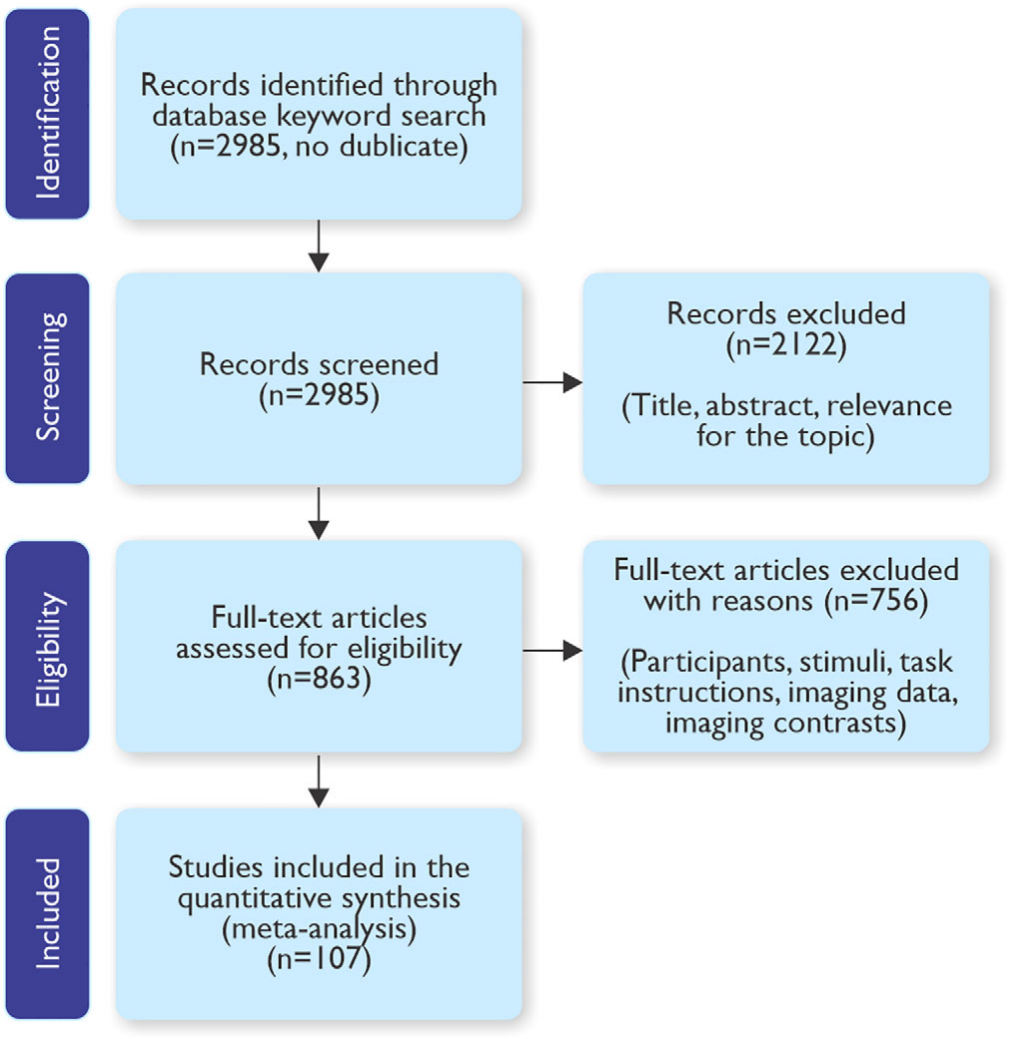
PRISMA flow chart of the study search and study selection process

Potentially eligible studies for perceptual decision‐making of emotions were identified by conducting a search on PubMed for studies published online between January 1st 1989 and July 1st 2019, using the following keyword combinations: (fmri OR pet) AND (emotion* OR affective) AND (face* OR facial OR body OR posture* OR voice* OR vocal). Study inclusion was restricted to whole‐brain functional magnetic resonance imaging studies or PET studies written in English on perceptual decision‐making on emotions across a variety of tasks. A series of five inclusion and exclusion criteria were further applied at the level of participants, stimuli, task instructions, imaging data, and imaging contrasts reported. All potential studies were independently screened by each author, and were selected if they had sufficient experimental and data quality. A flow chart of the study search, inclusion and exclusion criteria, and the final selection of studies is shown in Figure 2.

### 2.2 Participants selection

Only healthy adults with a median or average age between 18 and 59 years old were included in the analyses. Studies with less than eight participants were excluded, as they would mostly introduce noise in the data (Eickhoff et al., 2009). Clinical and pharmaceutical studies were included if they reported separate, within‐group analyses for the controls or the placebo condition. Studies on imaging genetics were included if they randomly selected the sample out of the general population and allowed the allele quotas to fall out naturally.

### 2.3 Experimental stimuli

The included studies used emotional expressions conveyed in the face, voice, or body postures according to either a basic emotions model (i.e., anger, fear, joy, sadness, disgust, surprise) or a valence‐arousal model (e.g., mildly/highly pleasant/unpleasant). Emotional expressions were either prototypical or morphed/filtered; unimodal (e.g., faces only, voices only) or multimodal (e.g., faces and voices together); presented either in a static or dynamic form. Furthermore, the emotional stimuli must have been previously validated as pertaining to an emotional construct (e.g., Ekman faces) or must have been validated in a pilot study specific for the paradigm in question. For the sake of uniformity, one study using point‐light stimuli to depict emotional faces and body expressions was not included (Atkinson, Vuong, & Smithson, 2012). Mental states (e.g., Mind in the eye task) and sexual or erotic stimuli were also excluded.

### 2.4 Task instructions

Because we were interested in the perceptual decision‐making on emotional expressions, we only included studies that used a paradigm of active deliberation over perceived emotional expressions (e.g., identify, categorize, and discriminate). We specifically excluded studies that prompted the participants to feel the emotion perceived or to react to it, as well as studies looking into learning (e.g., fear conditioning), memory for emotional stimuli (e.g., recall of happy vs. neutral faces) or the effects of emotion on cognition. Similarly, studies that required emotional expressions to be imagined, anticipated, or generated were excluded. Studies on backward masking of emotions (i.e., an emotional face presented at a near‐threshold detection rate, e.g., 67 ms) and binocular rivalry (e.g., emotional faces superimposed on houses) were included only if the participants were fully aware of the stimuli.

### 2.5 Imaging data

The brain activation data must have been reported in either the standard MNI or Talairach space. Studies failing to report the imaging space were not included. Furthermore, only studies on changes in regional activation (i.e., as revealed by task comparison or by image subtraction method) were included. Because the activation likelihood estimation (ALE) meta‐analysis has been validated with contrast‐based analyses, we excluded data on changes in functional or effective connectivity, and data reporting an interaction between stimulus and time, or task and time. Similarly, we excluded studies reporting contrast‐based deactivation, as it is conceptually recommended that activation and deactivation studies are investigated separately (Müller et al., 2018).

### 2.6 Imaging contrasts

We were particularly interested in an “emotion versus neutral” contrast within each type of perceptual decision task. Therefore, we primarily included studies reporting either a main effect of emotion (irrespective of the type of emotion, e.g., all emotions vs. neutral; irrespective of modality, e.g., emotional faces and voices vs. neutral faces and voices) or simple effects of emotion (e.g., discriminate happy vs. discriminate neutral, when other emotions were also reported). Studies were also included if they reported a main effect of task (e.g., discriminate emotional faces vs. discriminate geometrical shapes). We specifically excluded contrasts using resting‐state or fixation cross as a baseline for comparison or contrasts comparing various emotions against each other. Finally, we excluded imaging contrasts correlating with other attributes (e.g., anxiety, personality traits).

Following the keyword search, 3,278 studies were highlighted. The selection process of these articles took place in two stages. First, the titles and abstracts were assessed, and the articles were retrieved based on for relevance. Second, the full text of relevant articles was assessed to determine whether the five inclusion and exclusion criteria were met (i.e., participants selection, experimental stimuli, task instructions, imaging data, and analysis contrasts). Following this procedure, 3,278 initial publications were found, of which 107 articles (111 experiments) fulfilled all inclusion and exclusion criteria.

### 2.7 Post hoc classification of paradigms

As with any meta‐analysis, it is imperative that the individual tasks within a target paradigm are as similar as possible, that is, that task heterogeneity is reduced as much as it is theoretically possible. Following the identification of the 107 eligible articles but before we conducted the meta‐analyses, we performed a post hoc classification of the perceptual decisional tasks on emotions to identify homogenous paradigms. In grouping the eligible studies, we generally ignored the authors' nomenclature as it tended to be heterogeneous and inconsistent. For example, discriminating an emotional expression (i.e., comparing two stimuli against each other on a particular dimension) was sometimes referred to as “detection” (Buchanan et al., 2000), “judgment” (Critchley et al., 2000), or “identification” (Gur et al., 2007). Similarly, labeling emotional expressions was termed “discrimination” (Johnston, Mayes, Hughes, & Young, 2013; Kotz et al., 2003), “categorization” (Pichon, de Gelder, & Grèzes, 2009; van de Riet, Grèzes, & de Gelder, 2009), “recognition” (Derntl et al., 2012), “identification” (Kitada, Johnsrude, Kochiyama, & Lederman, 2010), “classification” (Szameitat et al., 2010), or even “comprehension” (Alba‐Ferrara, Hausmann, Mitchell, & Weis, 2011). Given the high variety with which the nomenclature was used, we opted to classify the tasks based on the similarity in the instructions. We then gave these formed classes of tasks new labels that would fit with the corresponding paradigm.

**Figure 3:**
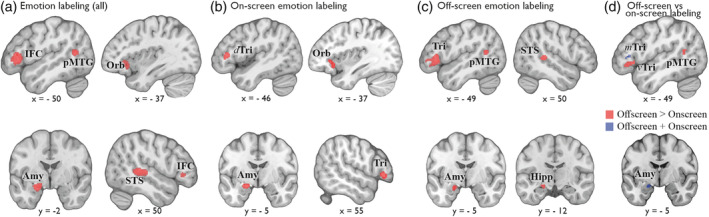
Results of the individual neuroimaging meta‐analysis on the paradigm of labeling of emotional expressions when the available choice labels are (a) displayed on the screen or (b) not displayed on the screen. (c) Results of the contrast analysis between the meta‐analysis on off‐screen emotion labeling and (d) on‐screen emotion labeling. Amy, amygdala; *d*Tri, dorsal pars triangularis; Hipp, hippocampus; IFC, inferior frontal cortex; *m*Tri, middle pars triangularis; Orb, pars orbitalis; pMTG, posterior middle temporal gyrus; STS, superior temporal sulcus; *v*Tri, ventral pars triangularis

A starting point in our post hoc classification was the seminal study by Hariri et al. (2002) who found that a nonverbal emotion‐matching task greatly activates the bilateral amygdala. In this task, participants must match the facial expression (usually angry or fearful) of one of two faces to that of a simultaneously presented target expression. Since its implementation, this paradigm has been increasingly used in research on facial expressions in both healthy and clinical populations. Furthermore, Burklund, Craske, Taylor, and Lieberman (2015) argued that emotion matching and emotion labeling tap into distinct brain networks. Given the high possibility that amygdala activation could be driven mostly by the nonverbal matching task, which might not be characteristic to other forms of perceptual decisional tasks, we considered *emotion matching* and *emotion labeling* as distinct classes of decision‐making (Figure 3). We grouped studies on emotion labeling to include those paradigms where participants are asked to associate a perceived emotional expression with an appointed emotion label. Specifically, emotion labeling included forced‐choice studies with response buttons dedicated to each emotional label or emotional construct. Based on task instructions, emotion labeling could be further divided into *off‐screen labeling* and *on‐screen labeling*, with the latter presenting the target emotional expression along with simultaneous verbal descriptions of the possible choice labels (i.e., on‐screen), and the former requiring participants to perform without such visual aids (i.e., off‐screen). Such a distinction in task instructions might prompt participants to use different cognitive strategies to perceive the emotions (Figure 3).

Additionally, we grouped experiments into *emotion discrimination* and *emotion rating*. *Discrimination* incorporates experiments in which participants compare a target stimulus against background noise (e.g., detect the presence or absence of a stimulus) or against other similar stimuli (e.g., are these stimuli the same or different?) and it draws inspiration from classical psychophysical studies (e.g., Phillips, Channon, Tunstall, Hedenstrom, & Lyons, 2008; Soliunas & Gurciniene, 2007; Van Hout, Hautus, & Lee, 2011; Figure 3). *Emotion rating* consists of studies in which participants must gauge the intensity or the valence of the emotional expression on a given scale and draws inspiration from neuropsychological studies with brain‐lesioned patients (e.g., Adolphs et al., 2000; Fruhholz & Staib, 2017; Figure 2).

### 2.8 Coordinate‐based meta‐analyses

Following the post hoc classification of studies into paradigms based on task instructions, we proceeded with separate meta‐analyses on each paradigm of perceptual decisions on emotions. We opted for the coordinate‐based ALE meta‐analysis, which identifies brain areas of convergent neural activity across different experiments, empirically determining whether this clustering is greater than expected by chance. The ALE algorithm, as implemented in the latest version of GingerALE 2.3.6 (http://brainmap.org/ale), captures the spatial uncertainty associated with reported coordinates, treating them as the centers for 3D Gaussian probability distributions (Turkeltaub, Eden, Jones, & Zeffiro, 2002) with widths based on empirical between‐subject and between‐template comparisons (Eickhoff et al., 2009). One modeled activation (MA) map is then created for each experiment by merging the probability distributions of all activation foci (Turkeltaub et al., 2012). If more than one focus from a single experiment is jointly influencing the MA map, then the maximum probability associated with any focus reported by the given experiment is used. Voxel‐wise ALE scores (union across these MA maps) then quantify the convergence across experiments at each location in the brain. As functional activations occur predominantly in gray matter areas, ALE scores are computed only for voxels with more than 10% probability of containing gray matter (Evans, Collins, & Milner, 1992). The resulting random‐effects inference focuses on the above‐chance convergence across studies rather than the clustering within a particular study (Eickhoff et al., 2009). To distinguish “true” from random convergence, ALE scores are compared to an empirical null distribution reflecting a random spatial association among all MA maps.

A major focus of the present study was to determine the differences and similarities in brain structures between the different types of perceptual decisions on emotions. In this regard, we performed a series of conjunction and contrast analyses between the different types of decisions on emotions. We have to note that the number of studies classified to certain paradigms (see above) differed across these paradigms (Table 1). However, GingerALE accounts for an unbalanced number of studies that are subjected to certain contrasts by means of data simulation and permutation (Eickhoff et al., 2011). Thus, the brain activations resulting from these comparisons between paradigms are very unlikely influenced by the study number.

**Table 1:** Summary of the emotion perception paradigms included in the meta‐analyses

Perceptual task	Articles	Experiments	Participants	Average no. of participants	Foci
Emotion labeling	45	47	1,089	23	723
Off‐screen	20	20	486	25	282
Onscreen	25	27	603	22	441
Emotion matching	34	35	853	24	528
Emotion discrimination	18	19	290	15	183
Emotion rating	10	10	218	22	173

The conjunction analysis is computed using the conservative minimum statistic inference (Nichols, Brett, Andersson, Wager, & Poline, 2005), which calculates a simple overlap between regions that were found statistically significant in the individual meta‐analyses. This implies that only those regions that are significant on a corrected level in both individual meta‐analyses are considered. Contrast analyses are performed by computing the voxel‐wise difference between two ensuing ALE maps (Eickhoff et al., 2011). Specifically, all experiments contributing to either the minuend or the subtrahend in these contrast analysis are then pooled and randomly divided into two groups of the same size as the two original sets of experiments reflecting the contrasted ALE analyses. ALE scores for these two randomly assembled groups are calculated and the difference between these ALE scores is recorded for each voxel in the brain. Repeating this process several thousand times yields an expected distribution of ALE‐score differences under the assumption of exchangeability. The “true” difference in ALE scores is tested against this null‐distribution yielding a posterior probability that the true difference was not due to random noise in an exchangeable set of labels. The resulting probability values are then thresholded and inclusively masked them by the respective main effects, that is, the significant effects of the ALE analysis for that condition. A correction for multiple comparisons is not applied to the contrasts analyses because GingerALE restricts the search space to voxels that had survived the threshold in the main effect for the minuend (Eickhoff et al., 2011).

The GingerALE algorithm uses two sets of statistical corrections (or thresholds). The first correction represents the *p* value that a voxel must surpass to be considered (i.e., cluster‐forming threshold) while the second correction specifies the number of contiguous voxels that must simultaneously surpass cluster‐forming threshold to be considered a significantly active cluster of voxels (i.e., cluster‐level family‐wise error corrected thresholding). The reasoning for this dual‐threshold correction is that voxels representing false alarms due to noise are more likely to be randomly distributed throughout the brain and thus much less likely to occur in contiguous groups of voxels than in single voxels (Eickhoff, Bzdok, Laird, Kurth, & Fox, 2012; Lieberman & Cunningham, 2009). We thresholded individual meta‐analyses with a cluster‐forming threshold at voxel level *p* < .001 and a cluster‐level family‐wise error corrected thresholding of *p* < .01 (Eickhoff et al., 2012). To determine null‐distributions, we conducted 10,000 repetitions. Contrasts analyses were based on the subtraction of single dataset meta‐analyses and the results were thresholded at *p* < .05 (i.e., 5% probability that the differences observed between datasets are due to random noise). To assess the null distribution, we again opted for 10,000 repetitions. A correction for multiple comparisons is not applied to the contrasts analyses because GingerALE restricts the search space to voxels that have survived the threshold in the main effect for the minuend (Eickhoff et al., 2011). To ensure enough statistical power, we limited our analyses to data sets which contained at least 17 experiments (Eickhoff et al., 2016). All meta‐analyses results were localized and labeled using the Yale BioImage Suite's digital medical atlas (http://bioimagesuiteweb.github.io/webapp; Papademetris et al., 2006), and visualized using the MRIcron software (http://nitrc.org/projects/mricron) with the MNI brain template.

## 3 RESULTS

A summary of the post hoc classification of eligible studies can be seen in Table 1. Most of the studies used various tasks of emotion labeling, resulting in 47 experiments, 1,089 participants and 723 distinct activation coordinates in the brain (foci), followed by 35 experiments of emotion matching, 19 experiments of emotion discrimination, and 10 experiments of emotion rating (Table 1).

### 3.1 Individual meta‐analyses of decisional tasks

Neuroimaging meta‐analyses as those calculated by the GingerALE software reveal the most consistently reported neural activity for a given paradigm, for example, emotion labeling. In other words, they reveal the brain structures most regularly engaged during a paradigm, above, and beyond individual differences in experimental design, settings, or stimuli.

The paradigm of emotion labeling was characterized by extensive activation in the left IFC (pars triangularis and pars orbitalis), as well as the left amygdala, the right superior temporal sulcus, the left posterior middle temporal gyrus (MTG) were recruited and the right pars triangularis of the IFC (Table 2, Figure 3a). When the available choice options were displayed on the screen, emotion labeling was associated with a strongly lateralized recruitment of the left amygdala and several patches along the left IFC, namely mid and dorsal pars triangularis, and pars opercularis (Table 2, Figure 3b). When the available choice options were not displayed on the screen for the duration of the trials, converging brain activity was found in the left mid and ventral pars triangularis, the left posterior MTG, the left amygdala extending into the hippocampus, and the right superior temporal sulcus (Table 2, Figure 3c).

**Table 2:** Brain regions with significant convergence of activity pertaining to each paradigm of emotion perception

Paradigm	Anatomical structure	x	y	z
Emotion labeling (all)^a^^,^ e	Cluster 1 (k = 4,720)			
	L mid pars triangularis (IFC)	−48	26	4
	L dorsal pars triangularis (IFC)	−46	34	4
	L pars orbitalis (IFC)	−40	22	−8
	L pars orbitalis (IFC)	−32	26	−4
	L pars orbitalis (IFC)	−46	36	−10
	Cluster 2 (k = 2,632)			
	R superior temporal sulcus	52	−36	4
	R superior temporal sulcus	58	−50	8
	Cluster 3 (k = 2,280)			
	L amygdala	−20	−4	−16
	Cluster 4 (k = 920)			
	L posterior middle temporal gyrus	−50	−58	12
	L posterior middle temporal gyrus	−56	−50	6
	Cluster 5 (k = 608)			
	R pars triangularis (IFC)	54	30	−2
On‐screen labeling^b^^,^ e	Cluster 1 (k = 992)			
	L amygdala	−20	−2	−16
	Cluster 2 (k = 760)			
	L pars orbitalis (IFC)	−38	22	−8
	L pars orbitalis (IFC)	−34	28	−2
	Cluster 3 (k = 712)			
	L mid pars triangularis (IFC)	−46	32	8
	Cluster 4 (k = 488)			
	R ventral pars triangularis (IFC)	56	28	0
Off‐screen labeling^c^^,^ e	Cluster 1 (k = 4,320)			
	L mid pars triangularis (IFC)	−48	26	4
	L ventral pars triangularis (IFC)	−50	16	−4
	L mid pars triangularis (IFC)	−46	38	0
	Cluster 2 (k = 1,344)			
	L hippocampus	−20	−12	−14
	L amygdala	−20	−8	−16
	Cluster 3 (k = 1,016)			
	L posterior middle temporal gyrus	−48	−56	14
Emotion matching^e^	Cluster 1 (k = 3,312)			
	R amygdala	22	−4	−18
	Cluster 2 (k = 3,216)			
	L amygdala	−22	−6	−18
	Cluster 3 (k = 3,128)			
	R inferior frontal junction	46	16	22
	R inferior frontal sulcus	52	26	22
	Cluster 4 (k = 2,696)			
	R visual association area	26	−96	−2
	Cluster 5 (k = 2,496)			
	L inferior frontal junction	−44	18	26
	Cluster 6 (k = 1,848)			
	R fusiform gyrus	40	−52	−24
	R fusiform gyrus	40	−64	−14
	Cluster 7 (k = 1,360)			
	L visual association area	−22	−96	−6
	Cluster 8 (k = 1,256)			
	L fusiform gyrus	−40	−54	−22
	Cluster 9 (k = 1,152)			
	L + R dorsomedial frontal cortex	−2	16	50
	Cluster 10 (k = 864)			
	L thalamus	−22	−30	−2
	Cluster 11 (k = 696)			
	R intraparietal sulcus	34	−56	46
	Cluster 12 (k = 656)			
	R superior temporal sulcus	58	−48	14
	R superior temporal sulcus	52	−44	10
	Cluster 13 (k = 536)			
	R thalamus	24	−30	0
	Cluster 14 (k = 488)			
	L intraparietal sulcus	−30	−56	44
Emotion discrimination^d^^,^ e	Cluster 1 (k = 1,264)			
	R pars opercularis (IFC)	42	16	20
	R pars opercularis (IFC)	52	22	24
	Cluster 2 (k = 864)			
	L amygdala	−24	0	−18
	L peri‐amygdala	−32	6	−22
Emotion rating^d^^,^ e	Cluster 3 (k = 1,152)			
	L amygdala	−26	2	−18
	L hippocampus	−32	−6	−20

*Note*: k = number of voxels in a cluster. L = left hemisphere. R = right hemisphere. IFC = inferior frontal cortex. x, y, z = coordinates of the peak brain activations in the MNI stereotaxic coordinate system.

a All tasks of labeling emotions from facial, vocal, and body expressions.

b Labeling emotions from facial, vocal, and body expressions when the choice options are displayed on screen.

c Labeling emotions from facial, vocal, and body expressions when the choice options are not displayed on screen.

d Activation revealed with a more lenient statistical threshold.

e Based on the contrast of emotional versus neutral expressions or a suitable control task at the study level.

**Figure 4:**
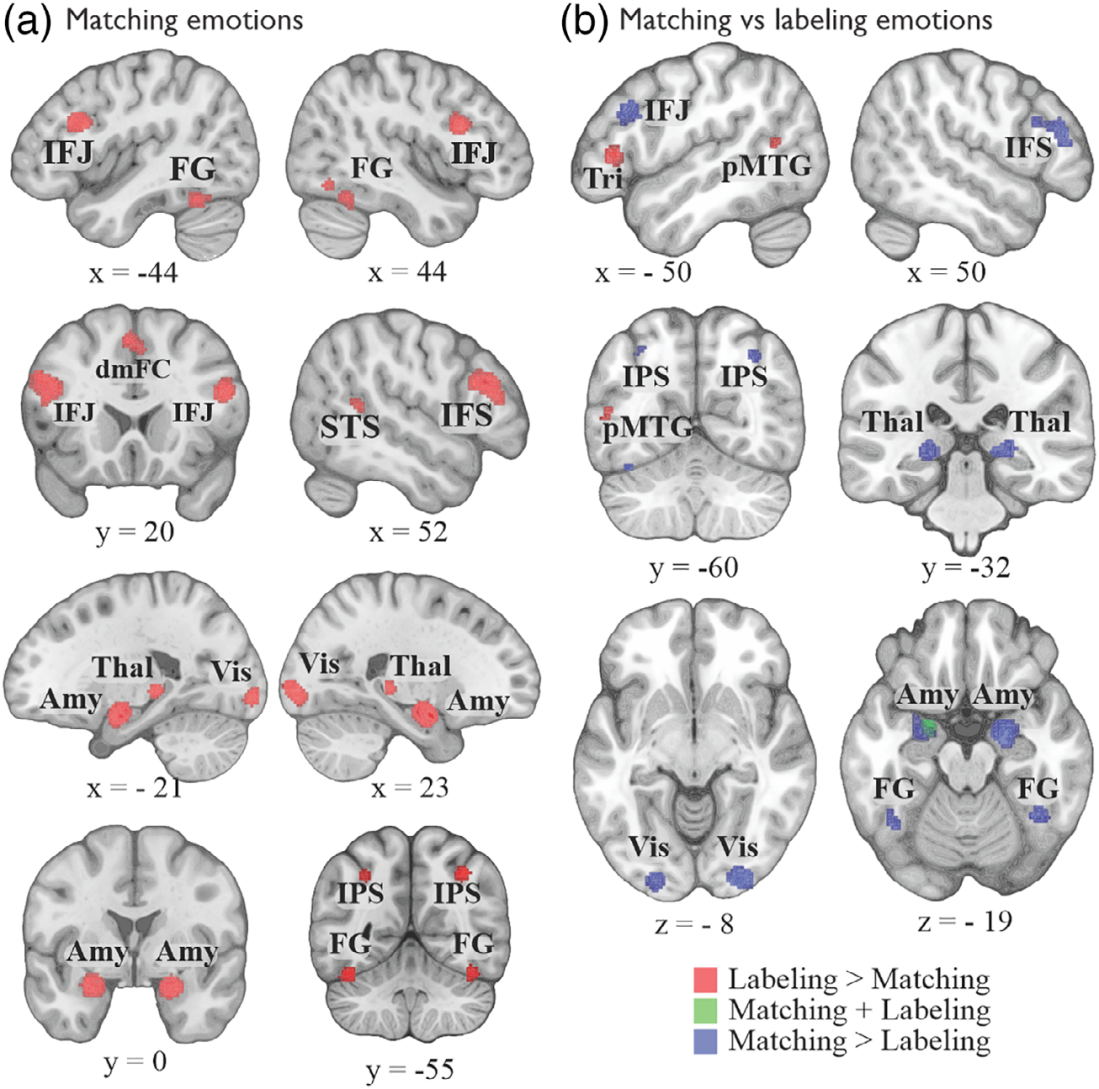
(a) Results of the individual neuroimaging meta‐analysis on emotion matching. (b) Results of the contrast analysis between the meta‐analyses on emotion matching and emotion labeling. Amy, amygdala; dmFC, dorsomedial frontal cortex; FG, fusiform gyrus; IFJ, inferior frontal junction; IFS, inferior frontal sulcus; IPS, intraparietal sulcus; pMTG, posterior middle temporal gyrus; STS, superior temporal sulcus; Thal, thalamus; Tri, pars triangularis; Vis, the visual cortex

**Figure 5:**
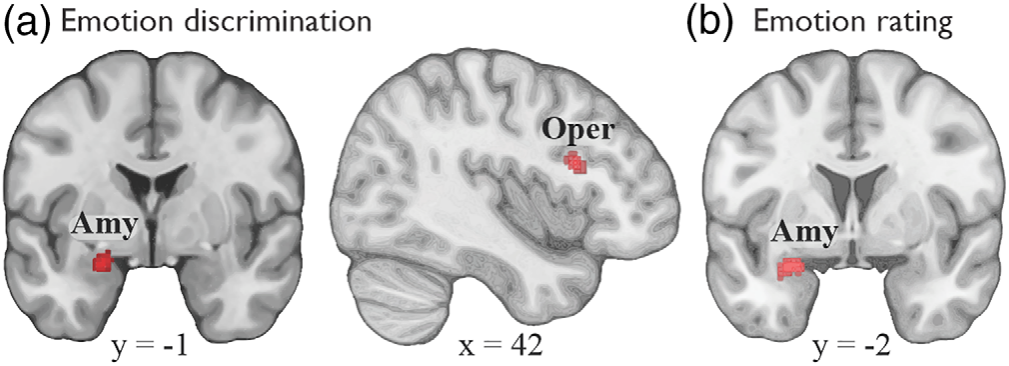
(a) Results of the individual neuroimaging meta‐analysis on emotion discrimination and (b) emotion rating. Amy, amygdala; Oper, pars opercularis of the inferior frontal cortex

The paradigm of nonverbal matching of emotional facial expressions is correlated with bilateral activations in the inferior frontal junction (IFJ), the amygdala, the visual association cortex, the fusiform gyrus, the dmFC, the intraparietal sulcus, and the left thalamus (Table 2, Figure 4a). At the chosen cluster‐forming threshold of *p* < .0001, no brain regions showed statistical convergence of neural activity for the paradigms of emotion discrimination and emotion rating. Due to the overall lower number of experiments pertaining to these two paradigms compared to emotion labeling and emotion matching, we opted to lower the cluster‐forming threshold to *p* < .001, which revealed that the left amygdala was associated with both emotion discrimination and emotion rating (Table 2, Figure 5a). At this more lenient threshold, the right pars opercularis of the IFC was additionally activated for emotion discrimination (Table 2, Figure 5a).

### 3.2 Contrast and conjunction analyses

Unlike individual neuroimaging meta‐analyses, which find the most regularly engaged brain structures during a given paradigm, contrast analyses compare thus found brain regions of engaged neural activity between two paradigms, that is, meta‐analysis of paradigm A versus meta‐analysis of paradigm B. In other words, a contrast analysis reveals which brain regions, if any, are more consistently more recruited by paradigm A *compared to* paradigm B. A conjunction analysis, on the other hand, reveals which regions, if any, are regularly recruited during paradigms A *and* B.

Comparing “off‐screen” against “on‐screen” emotion labeling revealed that the left ventral pars triangularis extending, the right superior temporal sulcus, and the left posterior MTG were more consistently recruited during off‐screen labeling, that is, when available choice options are not displayed on the screen during the perception decision‐making process (Table 3, Figure 3d). Contrasting “on‐screen” emotion labeling with “off‐screen” labeling did not reveal any significant brain activation that is more consistently reported by the former compared to the latter. Last, the conjunction analysis showed that activations in the left amygdala and the left mid pars triangularis of the IFC were common for both onscreen and off‐screen labeling of emotions (Table 3, Figure 3d).

**Table 3:** Contrast and conjunction analyses of paradigms of perceptual decisions on emotions

Analysis	Anatomical structure	*x*	*y*	*z*
On‐screen labeling^a^ vs. off‐screen labeling^b^	No brain regions with significant convergence of activity	—	—	—
Off‐screen labeling^b^ vs. on‐screen labeling^a^	Cluster 1 (*k* = 696)			
	L ventral pars triangularis (IFC)	−48	30	−8
	L ventral pars triangularis (IFC)	−50	22	−4
	Cluster 2 (*k* = 240)			
	L posterior middle temporal gyrus	−48	−52	16
	L posterior middle temporal gyrus	−46	−54	10
Off‐screen labeling^b^ and on‐screen labeling^a^	Cluster 1 (*k* = 240)			
	L amygdala	−20	−8	−16
	Cluster 2 (*k* = 144)			
	L mid pars triangularis (IFC)	−48	28	6
Label emotions^c^ vs. Match emotions^d^	Cluster 1 (*k* = 1,232)			
	L mid pars triangularis (IFC)	−51	29	4
	L mid pars triangularis (IFC)	−48	32	2
	L mid pars triangularis (IFC)	−44	30	4
	Cluster 2 (*k* = 312)			
	L posterior middle temporal gyrus	−54	−58	12
Match emotions^d^ vs. Label emotions^c^	Cluster 1 (*k* = 2,488)			
	R visual association areas	30	−94	−6
	R visual association areas	26	−94	−8
	Cluster 2 (*k* = 2,424)			
	R inferior frontal sulcus	54	32	20
	R inferior frontal junction	46	11	24
	Cluster 3 (*k* = 2,256)			
	R amygdala	20	−9	−18
	R amygdala	20	−4	−22
	Cluster 4 (*k* = 1,744)			
	L inferior frontal sulcus	−47	20	31
	Cluster 5 (*k* = 1,048)			
	L visual association areas	−22	−91	−9
	Cluster 6 (*k* = 1,040)			
	L amygdala	−28	0	−22
	L amygdala	−24	−6	−18
	Cluster 7 (*k* = 936)			
	R fusiform gyrus	44	−56	−24
	R fusiform gyrus	38	−54	−22
	Cluster 8 (*k* = 800)			
	L fusiform gyrus	−42	−60	−22
	L fusiform gyrus	−40	−52	−26
	Cluster 9 (*k* = 792)			
	L thalamus	−18	−28	−2
	L thalamus	−22	−32	0
	Cluster 10 (*k* = 696)			
	R intraparietal sulcus	34	−58	42
	R intraparietal sulcus	30	−56	42
	Cluster 11 (*k* = 536)			
	R thalamus	16	−32	−2
	Cluster 12 (*k* = 208)			
	L intraparietal sulcus	−32	−64	46
Label emotions^c^ c and Match emotions^d^	Cluster 1 (*k* = 1,664)			
	L amygdala	−22	−2	−16

*Note: k* = number of voxels in cluster. L = left hemisphere. R = right hemisphere. IFC = inferior frontal cortex. x, y, z = coordinates of the peak brain activations in the MNI stereotaxic coordinate system.

a Labeling emotions from facial, vocal, and body expressions when the choice options are displayed on screen.

b Labeling emotions from facial, vocal, and body expressions when the choice options are not displayed on screen.

c All tasks of labeling emotions from facial expressions alone.

d Matching emotions from facial expressions.

The paradigm of emotion matching relies exclusively on facial expressions. Therefore, to ensure a fair comparison, we looked at the similarities and differences between this paradigm and a subset of emotion labeling, that is, labeling facial expressions. Compared to the nonverbal task of emotion matching, the verbal labeling of emotional facial expressions distinctly recruits the left pars triangularis, the right superior temporal sulcus, and the left posterior MTG (Table 3, Figure 4b). Comparing emotion matching against emotion labeling revealed an extended network of bilateral brain regions comprising of the bilateral visual association areas, amygdalae, the IFJ, the dorsal medial frontal cortex, the fusiform gyri, the intraparietal sulci, and the thalami that were uniquely recruited during emotion matching (Table 3, Figure 4b). The conjunction analysis revealed that emotion matching and emotion labeling of facial expressions similarly recruited the left amygdala (Table 3, Figure 4b).

Due to the insufficient number of experiments for emotion discrimination and emotion rating (Eickhoff et al., 2016), we were unable to formally run contrast and conjunction analyses between these paradigms and emotion labeling and emotion matching.

## 4 DISCUSSION

The present study tried to address the existing gaps in the literature of emotion perception, namely the general lack of acknowledgment concerning the heterogeneity of perceptual tasks (Elliott et al., 2011; Ong et al., 2015) and how the brain idiosyncratically computes the various perceptual decisions on emotions. We opted for an evidence‐based approach, based on the existing literature about the neurocognitive mechanisms behind our perceptual decisions on others' emotions.

In this regard, we reviewed the neuroimaging literature of emotion perception spanning 30 years. This resulted in three major observations. First, there are largely four research paradigms used to investigate perceptional decision‐making on emotions, referred to in this article as emotion labeling, matching, discrimination, and rating. Second, each paradigm of decision‐making on emotions tapped into different neural structures that reflect the putative cognitive demands of the decision‐making task at hand. Third, the left amygdala was responsive across all classes of decisional paradigms, regardless of task instructions, clarifying the degree of involvement of the amygdala in the explicit evaluation of emotions. In the following paragraphs, we will discuss these findings and we will conclude by proposing a neurocognitive model of perceptual decision‐making on emotions that outlines the information flow in the brain needed for a proper understanding of other individuals' emotions.

### 4.1 Emotion matching

Nonverbally matching emotional expressions, such as matching a target emotional expression to various other expressions perceived, recruits brain regions of sensory processing regions and of higher cognition. The presence of sensory regions is understandable given the nature of the control task used at the level of individual experiments. The control task invariably involves matching geometrical shapes based on low‐level perceptual cues. Contrastingly, the facial expressions in the matching task contain almost exclusively emotional faces (i.e., no neutral faces), most often fearful and angry. The comparison between matching highly aroused emotional faces and matching geometrical shapes would thus reveal residual activation in sensory brain regions involved with processing the complex perceptual cues found in emotional expressions. These sensory processing regions involved bilateral visual association areas, fusiform gyrus, thalamus, and intraparietal sulci, which have all been implicated in processing human faces (Arcurio, Gold, & James, 2012; Frühholz, Fehr, & Herrmann, 2009; Haxby et al., 2000, 2002; Rossion & Retter, 2015; Yovel, 2016).

The emotion‐matching paradigm also strongly recruited the bilateral amygdala. Generally thought of as a module of automatic detection of emotions (Frühholz & Grandjean, 2013a; Öhman, 2002; Pannese et al., 2015, 2016; Phelps & LeDoux, 2005; Vuilleumier, Armony, Driver, & Dolan, 2001), the amygdala is preferentially recruited by angry and fearful faces (Adams, Gordon, Baird, Ambady, & Kleck, 2003; Milesi et al., 2014; Phelps et al., 2001; Repeiski, Smith, Sansom, & Repetski, 1996), which are the predominant stimuli in the emotion‐matching paradigm. However, an alternative role for involvement of the amygdala is not as an automatic detector of emotions per se, but rather as a detector of relevant and salient stimuli, of which emotional expressions represent a subclass (Sander et al., 2003). For example, the amygdala responds to novel neutral faces (Schwartz, Wright, Shin, Kagan, & Rauch, 2003) and neutral faces of a different race (Hart et al., 2000) and abstract figures with learned associations with food rewards (Gottfried, O'Doherty, & Dolan, 2003). Adding an identity‐matching paradigm involving neutral faces only, Wright and Liu (2006) showed that bilateral amygdala activity was responsive during both identity and emotion‐matching tasks. Thus, it seems that the matching task itself triggers amygdala activity. The authors argued that the matching task adds relevance to the stimuli, including neutral faces. Viewed alone, neutral faces would be expected to have less inherent relevance than emotional faces but, during a matching task, they must acquire task‐related relevance. That is, out of the two possible faces to be matched, one becomes the “right” face while the other becomes the “wrong” face.

In addition to sensory processing regions, we found additional activation in bilateral IFJ and the dmFC during the emotion‐matching paradigm. The IFJ is a structurally distinct region located at the junction of the inferior frontal sulcus (IFS) and the inferior precentral sulcus (Amunts et al., 2010). Lesion studies (Petrides, 1985, 2005), transcranial magnetic stimulation (Verbruggen, Aron, Stevens, & Chambers, 2010), and neuroimaging experiments (Clos, Amunts, Laird, Fox, & Eickhoff, 2013; Harding, Yücel, Harrison, Pantelis, & Breakspear, 2015; C. Kim, Cilles, Johnson, & Gold, 2012; Sundermann & Pfleiderer, 2012) have linked the IFJ to cognitive switching across a variety of domains such as context switching (e.g., shifting between task rules or cognitive rules), perceptual switching (e.g., switching attention between perceptual features of a stimulus or between stimuli) and response switching (e.g., switching between different stimulus‐response mappings). The dmFC is a complex structure that spans several distinct regions (Öngür, Ferry, & Price, 2003) and plays different roles in social cognition (Amodio & Frith, 2006; Dricu & Frühholz, 2016; J. P. Mitchell, Macrae, & Banaji, 2005; Schurz et al., 2014). We found that the same portion of the dmFC is consistently reported in nonverbal tasks of appraising the mental states of both human and nonhuman agents based on observable cues (Döhnel et al., 2012; Gallagher et al., 2000; J. W. Kim et al., 2005; Schlaffke et al., 2015; Schurz et al., 2014; Völlm et al., 2006). In the domain of emotions, the dmFC is highly active when judging the appropriateness of specific facial emotions in a given context (J. W. Kim et al., 2005) or inferring whether someone is genuinely sad/happy or is simply posing for the camera (McLellan, Wilcke, Johnston, Watts, & Miles, 2012). Furthermore, dmFC is also recruited when participants must reason about the most likely scenario that caused an emotional facial expression (Prochnow, Brunheim, Steinhauser, & Seitz, 2014).

It thus appears that the nonverbal task of emotion matching invites participants to simultaneously infer the putative mental states associated with the perceived emotional expression. Beyond perceptually attending to each facial expression (as indexed by the sensory regions and intraparietal sulci), it is reasonable to expect that participants also mentally switch from appraising the mental state associated with the target emotional expression to appraising the mental states of each of the two potential choice options. Upon encountering emotional expressions of others, we may automatically infer their traits and mental states, and spontaneously integrate this information into our impressions about others (Uleman et al., 1996; Van Overwalle, 2009, 2011). Strong functional (Harding et al., 2015; Sundermann & Pfleiderer, 2012) and structural connectivity (Ford, McGregor, Case, Crosson, & White, 2010; Sallet et al., 2013) between the dmFC and the IFJ suggests that these higher cognitive brain structures work together to coordinate decoding emotions and inferring mental states in order to perform the matching task.

### 4.2 Emotion labeling

We found a strongly left lateralized pattern of activation during all tasks of emotion labeling (perceiving expressions from the face, voice, or body posture and associating them with a label pertaining to an emotion construct, such as joy or fear). This lateralization is very much in line with relevant lesion (Bates et al., 2001; Riès, Dronkers, & Knight, 2016) and neuroimaging literature (Vigneau et al., 2006) on language production and comprehension. However, we did not find converging neural activation in sensory processing brain regions. Unlike the emotion matching paradigm, the control task used in emotion labeling paradigms often matches the emotional expressions in perceptual complexity. In fact, 35 out of the 45 experiments used neutral faces, voices, or body postures as a baseline. Therefore, activations in brain regions associated with mental processes such as basic perception, attention, and working memory would have been canceled out.

When comparing results for emotion labeling tasks that either displayed or hid possible response labels alongside the stimuli (“on‐screen” vs. “off‐screen” labeling), we found some similarities and several distinctions. Specifically, the left mid pars triangularis of the IFC and the amygdala were similarly recruited during “on‐screen” and “off‐screen” emotion labeling. Putatively, what “on‐screen” and “off‐screen” labeling share in terms of cognitive processing is the successful retrieval of semantic knowledge about the world around us, including emotional constructs (Hamberger, Habeck, Pantazatos, Williams, & Hirsch, 2014; Miceli, Amitrano, Capasso, & Caramazza, 1996; Raymer et al., 1997). Not surprisingly, the left mid pars triangularis has been implicated in the domain‐general access and retrieval of information from semantic memory (Costafreda et al., 2006; Gennari, MacDonald, Postle, & Seidenberg, 2007; Nee et al., 2013; Riès et al., 2016; Snyder, Banich, & Munakata, 2011).

The right ventral and left mid pars triangularis of the IFC were active during on‐screen labeling of emotions (when the available choice options are displayed on the computer screen for the duration of the trial simultaneously with the target emotional expression). Both of these regions are important in phonological coding (Adair, Schwartz, Williamson, Raymer, & Heilman, 1999; C. J. Price, 2010), a crucial cognitive process in single‐word reading during which letter‐to‐sound associations stored in long‐term memory are accessed and manipulated (Bokde, Tagamets, Friedman, & Horwitz, 2001; Palmer, 2000). Structural and functional differences in bilateral pars triangularis can distinguish between normal readers and individuals with dyslexia (Eckert et al., 2003; Leonard, Eckert, Given, Virginia, & Eden, 2006; Norton et al., 2014; Partanen, Siege, & Giaschi, 2018; Robichon, Levrier, Farnarier, & Habib, 2000). More importantly, training individuals with dyslexia on reading increases feedback connectivity from the left pars triangularis to the right pars triangularis and sensory cortices, which predicts the subsequent increased performance in reading speed (Frye, Wu, Liederman, & McGraw Fisher, 2010; Z. V. Woodhead et al., 2013). Together, this evidence points to the concerted effort of the left and right pars triangularis in the rapid and automatic reading of printed words, such as the ones displayed during the on‐screen labeling of emotions.

On‐line labeling of emotions also recruited the left pars orbitalis of the IFC. This region is consistently involved in semantic judgments on a wide range of stimuli, including single printed words (e.g., “is this word concrete or abstract?”; Cutting et al., 2006; Devlin, Matthews, & Rushworth, 2003; Fisher, Cortes, Griego, & Tagamets, 2012; Mainy et al., 2008; Poldrack et al., 1999; Z. Woodhead et al., 2012; Yvert, Perrone‐Bertolotti, Baciu, & David, 2012) and pairs of printed words (e.g., “are the two words semantically related?”; Booth et al., 2006; Gough, Nobre, & Devlin, 2005; Kemmerer, Rudrauf, Manzel, & Tranel, 2012; Kotz, Cappa, von Cramon, & Friederici, 2002; Liu et al., 2013; Mechelli, Josephs, Lambon Ralph, McClelland, & Price, 2007). More relevant to the task of on‐screen labeling, activity in the left pars orbitalis increases when participants match pictures to auditory labels based on semantic similarity (Schmithorst, Holland, & Plante, 2007). This finding strongly suggests that, upon independently perceiving the emotional expression of the stimulus and covertly reading the displayed emotion labels (as indexed by the left and right pars triangularis), participants perform the on‐screen emotion labeling task by semantically matching the emotional expression to the appropriate label.

Off‐screen emotion labeling recruited the left mid and ventral pars triangularis, left posterior MTG, and the right posterior superior temporal sulcus (pSTS). The left mid pars triangularis was also present in on‐screen labeling of emotions and it likely reflects accessing semantic knowledge (Costafreda et al., 2006; Gennari et al., 2007; Nee et al., 2013; Riès et al., 2016; Snyder et al., 2011). The ventral portion of the left pars triangularis has been implicated in verbal working memory (Rottschy et al., 2012), suggesting that off‐line labeling of emotions required maintaining the possible choice labels in working memory for the duration of the trials. The activation in the left posterior MTG coincides with a region long implicated in anomic aphasia (Foundas, Daniels, & Vasterling, 1998; Goodglass, 1980; Kay & Ellis, 1987; Raymer et al., 1997), a brain disorder in which the patient knows what an object is and how to use it, and can accurately select it from a group of objects but cannot name the object (Goodglass, 1980). In other words, anomic patients have intact semantic knowledge of the perceived object but no longer have access to the lexical‐phonological representation in the phonological output lexicon (Butterworth, 1992; Levelt, 1992; Raymer et al., 1997). In an independent line of research, neuroimaging studies have also consistently implicated the same region in object naming (Cathy J Price, 2012; Watson, Cardillo, Ianni, & Chatterjee, 2013). The critical role of the left posterior MTG in naming objects might come from its strategic location between the ventral processing stream—important for object recognition—and the auditory and visual association cortex (C. J Price, 2012; Raymer et al., 1997).

The right pSTS has long been associated with recognizing and understanding purposeful action and movements (Allison, Puce, & McCarthy, 2000) and the basic understanding of intentions (Pelphrey & Morris, 2006). Extensive research has shown that this major sulcal landmark in the temporal lobe is sensitive to gaze orientation (Hooker et al., 2003) and the movement of the human body (Kourtzi & Kanwisher, 2000; Saxe, Xiao, Kovacs, Perrett, & Kanwisher, 2004), hands (Gallagher & Frith, 2004; Holle, Obleser, Rueschemeyer, & Gunter, 2010) and face (Fruhholz, Godde, Lewicki, Herzmann, & Herrmann, 2011; Lee et al., 2010) through either direct perception (Allison et al., 2000) or implied movement (David & Senior, 2000). However, the pSTS is not sensitive to just any kind of movement, as significantly more activation occurs when people watch others perform complex versus simple actions (Castelli, Happé, Frith, & Frith, 2000), physically possible movements compared to impossible movements (Stevens, Fonlupt, Shiffrar, & Decety, 2000), meaningful versus meaningless movements (Decety et al., 1997; Schultz, Imamizu, Kawato, & Frith, 2004), and when people observe transitions between meaningful actions within a larger goal‐directed activity such as cleaning the kitchen (Zacks et al., 2001). Similarly, the pSTS sulcus is also involved when understanding and inferring actions from auditory cues alone, such as footsteps and action verbs (Bidet‐Caulet, Voisin, Bertrand, & Fonlupt, 2005; Hein & Knight, 2008). The overwhelming evidence suggests that the right pSTS is involved in decoding and understanding meaningful social actions conveyed by gaze direction, body movement, and other types of goal‐directed meaningful motion or implied by spoken words (see also Frith & Frith, 1999).

### 4.3 Emotion discrimination

When performing the meta‐analysis with a more lenient threshold, we observed activation in the left amygdala and the right pars opercularis during emotion discrimination, in which participants must compare a target stimulus against background noise (e.g., detect the presence or absence of a stimulus) or against other similar stimuli (e.g., are these stimuli identical or different?). The right pars opercularis has traditionally been involved in Go‐No Go tasks and Stop Signal Tasks, prompting its hypothesized recruitment in cognitive and behavioral control, and inhibition of prepotent tendencies (Aron, Robbins, & Poldrack, 2004; Derrfuss, Brass, Neumann, & von Cramon, 2005; Levy & Wagner, 2011). However, a recent reinterpretation of the literature has extended the involvement of the right pars opercularis to monitoring salient features such as identifying a target item or a target dimension in a series of nontargets or nondimensions (Hampshire, Duncan, & Owen, 2007; Hampshire, Thompson, Duncan, & Owen, 2008, 2009; Shallice, Stuss, Alexander, Picton, & Derkzen, 2008), and detecting prelearned target objects (Hampshire et al., 2007, 2008; Linden et al., 1999). Thus, it seems that, as with the other paradigms of emotion perception, emotion discrimination is associated with brain regions involved in general cognitive functions that are assumedly beyond rather basic perceptual tasks.

### 4.4 The role of the amygdala in emotion perception

The left amygdala was recruited across all tasks of explicit emotion perception, regardless of task instructions. This is an interesting finding since the degree of the amygdala's involvement in the explicit evaluation of emotions has been a contentious issue in affective neuroscience. On one hand, research suggests that the amygdala is recruited in specific types of perception such as implicit (e.g., Brück et al., 2011; LeDoux, 1998) or nonverbal (e.g., Hariri, Bookheimer, & Mazziotta, 2000) emotion perception. On the other hand, several lesion studies have pointed to a general role of the left amygdala in response to emotional faces (Markowitsch, 1999), voices (Frühholz et al., 2015), and pictures (Gläscher & Adolphs, 2003), a role that has been backed up by neuroimaging studies of emotion perception (Baas, Aleman, & Kahn, 2004; Sergerie, Chochol, & Armony, 2008) and emotion experience (Costanzo et al., 2015).

The left amygdala might be involved in the cognitive appraisal of emotional information, possibly via a global–local hemispheric bias (Baas et al., 2004; Cahill, 2003; Markowitsch, 1999) while the right amygdala might play an important role in the production of a general arousal level. Specifically, the right amygdala is more strongly engaged in fast global, albeit shallow, processing of emotional content (Henke, Landis, & Markowitsch, 1993; Morris, Öhman, & Dolan, 1999), while the left amygdala is more strongly engaged in active detailed processing (Krickl, Poser, & Markowitsch, 1987; Morris, Öhman, & Dolan, 1998). The preference for left but not right amygdala activation during emotion labeling could be argued that these tasks require an important degree of local and detailed processing. For example, participants might focus more on specific visuospatial details or acoustic features that differentiate an angry expression from a neutral expression to perform successfully. Conversely, emotion matching might depend on both coarse and detailed processing of emotional content, thus recruiting both the right and left amygdala. Systematic comparison of tasks that require global versus local processing of emotional material is needed via carefully designed experiments.

### 4.5 A neurocognitive model of perceptual decision‐making on emotions

By synthesizing the findings of the meta‐analyses (Figure 1b), we propose a multistage neurocognitive model that outlines the general flow of information from sensory processing regions to frontal brain regions (Figure 1c). We argue for three functional principles of perceptual decisions on others' emotions. First, brain regions in the left and right hemisphere are differentially recruited depending on whether the paradigm is verbal or nonverbal in nature. The verbal task of emotion labeling generously recruited multiple regions in the left hemisphere. These regions have long been implicated in language processing, including verbal working memory, access to semantic knowledge, reading, and naming (Goodglass, 1980; C. J. Price, 2010; Rottschy et al., 2012). The nonverbal task of emotion matching consistently recruited bilateral brain regions, resting on different cognitive mechanisms for optimal decision‐making. Instead of verbally processing the target emotion along with the two choice options in a similar way to off‐screen emotion labeling, our findings suggest that we match emotional expressions by primarily mentalizing about the perceived individuals' mental state during their episode of emotional expression. Second, the resulting information is made available to the IFC, where a functional anterior‐to‐posterior gradient emerged in the left hemisphere. While emotional information during the nonverbal paradigm of emotion matching converges in the most posterior parts (IFJ and IFS), emotion labeling recruited the middle pars triangularis and the anterior pars orbitalis. Third, the left amygdala was the only brain structure consistently recruited by all perceptual decisions, regardless of task instructions. Unlike the right amygdala, which is more strongly engaged in fast, global but shallow processing of emotional content (Henke et al., 1993; Morris et al., 1999), the left amygdala is more engaged in focal and detailed emotional processing (Frühholz, Trost, & Grandjean, 2014; Krickl et al., 1987; Morris et al., 1998; Pannese et al., 2015).

We must add a note concerning the possible limitations of our neurocognitive model. The model mostly hypothesizes feed‐forward mechanisms of information from sensory regions to higher‐level brain regions. Perceptual decision‐making, however, is unlikely a one‐way process, and also includes top‐down mechanisms of prediction based on context and prior expectations (e.g., Summerfield & De Lange, 2014). While we acknowledge the importance of these top‐down predictive mechanisms, the empirical evidence for the functional processes in emotion perception is surprisingly scarce. Some studies only recently began to empirically investigate these mechanisms (e.g., Bernstein & Yovel, 2015; Frühholz et al., 2016). Therefore, we refrain from explicitly incorporating such mechanisms in our current model given the limited empirical evidence on these functional and neural processes. Future studies should extend our current model by including top‐down processes of emotional predictions and expectations.

## 5 CONCLUSIONS, IMPLICATIONS, AND LIMITATIONS

When emotion perception consists of matching an expression against other expressions based on perceptual features (i.e., a novel facial expression against target facial expressions), we rely on a brain network that largely involves inferring the mental state underlying those expressions. Despite the artificial setting of such a paradigm, mentalization is present in many forms of emotion perception ranging from passive observation to explicit appraisal (Dricu & Frühholz, 2016; R. L. Mitchell & Phillips, 2015). On the other hand, when prompted to perceive and name the emotion in others, we rely on language processes to provide important support. One might argue that verbal perceptual decision‐making is synonymous with language processes, for example, relying on phonological coding and reading processes at large when the possible emotion labels are displayed on screen; maintaining emotion labels in our verbal working memory throughout the experiment when they are not displayed on screen; semantic matching between perceived expressions and emotion labels.

The present findings are partially compatible with two psychological theories of emotion. Strong appraisal theories suggest that we are capable of ascertaining the emotion of others by first inferring the appraisals associated with the perceived emotional expressions (Scherer et al., 2013; Scherer & Ellgring, 2007). Specifically, following sensory processing, we deduce the individual appraisals behind each pattern of facial muscle movements, body postures, or vocal prosody, and subsequently infer the emotion experienced by another individual. In this view, we should observe brain regions involved in “mindreading” (i.e., the process of inferring others' thoughts, beliefs, and desires) across all perceptual decisions on emotions (e.g., Frith & Frith, 2006; Perner & Esken, 2015). We found support for the strong version of appraisal theory in our nonverbal paradigm of emotion perception, that is, emotion matching, but not during emotion labeling or emotion discrimination. Conversely, constructivism theories—particularly the strong versions—give language a crucial role in perceiving (and, to some extent, inferring) one's own emotions and others' emotions (Barrett & Kensinger, 2010; Barrett et al., 2007; Lindquist & Gendron, 2013). In this view, brain regions pertaining to language processing should be present in all perceptual decisions on emotions. We found support for the constructivism theory of emotion in both the online and off‐line paradigms of emotion labeling, but not for emotion matching or emotion discrimination. We should mention, however, that off‐line labeling of emotions may still trigger a basic process of mental state inference, as suggested by the activation in the posterior superior temporal sulcus (Gergely & Csibra, 2003; Peelen, Atkinson, & Vuilleumier, 2010; Skerry & Saxe, 2014). Constructivism theories further claim that emotion perception is achieved via domain‐general cognitive processes that are not unique to emotional stimuli. We found support for this hypothesis in all the paradigms of emotion perception, including matching, labeling, and discrimination. For example, emotion matching is achieved via domain‐general processes of cognitive switching in the IFJ and eye gaze shifting in the intraparietal sulci (Clos et al., 2013; Corbetta, Patel, & Shulman, 2008). Emotion labeling is likely achieved via language processes that are not themselves unique to emotion, for example, phonological coding, semantic retrieval of emotion concepts (Rottschy et al., 2012; Schmithorst et al., 2007). Finally, emotion discrimination activates the right pars opercularis in a similar way to how discrimination between other classes of stimuli does (Hampshire et al., 2007, 2008; Linden et al., 1999).

In conclusion, strong appraisal and constructivism theories of emotion each partially predict the results in our meta‐analyses. As such, our findings align more readily with recent attempts at combining multiple elements from traditionally divergent accounts of emotion perception (Moors, 2017; Nesse, 2014). Instead of singling out some causal mechanisms of emotion perception at the expense of others, it may be helpful to acknowledge that the route to emotion inference is not singular and that there can be multiple mechanisms that differ in functionality and optimality. Our results further reiterate this by showing that emotion perception paradigms are, in fact, quite heterogeneous. Depending on task instructions, perceiving agents engage in different cognitive strategies that are called upon based on the situation. Clearly, more scientific progress can be achieved if predictions of emotion perception are tested on a larger scale of paradigms, beyond those included in this meta‐analyses that can only reflect the trends in the scientific community. On a more practical level, we strongly recommend that emotion researchers acknowledge the heterogeneity of emotion perception tasks, at the very least in its two variants, that is, verbal and nonverbal perception. Neuropsychologists have long used sophisticated batteries of tests that tap into various facets of emotion perception with the purpose of assessing subtle neurological damage, under the assumption that different brain regions underlie different perceptual tasks (Boller & Grafman, 2000; Wilhelm et al., 2014). However, the fields of psychology and neuroscience have yet to acknowledge the functional heterogeneity of emotion perception tasks. Furthermore, when bridging results across paradigms, authors should be aware that some emotion perception tasks are not directly comparable. Instead, more circumscribed extrapolation and interpretation of results is warranted.

We must note of course the limitations of our study. As meta‐analyses are based on the available empirical data, their results may be affected by scientific trends and a publication bias that disfavors null results. We tried to mitigate the publication bias in the literature by including neuroimaging results published in supplementary materials that were otherwise not reported in the main manuscripts. More importantly, as detailed elsewhere (Eickhoff & Bzdok, 2013; Rottschy et al., 2012), coordinate‐based meta‐analyses such as ours are less susceptible to publication bias than standard meta‐analytic approaches that examine effect sizes because the assessment of spatial convergence across experiments is not affected by additionally including (observed but unpublished) null results. We are therefore confident that the validity of our results was not substantially undermined by such bias.

Regarding scientific trends, our post hoc classification of emotion perception tasks resulted in four major paradigms, that is, labeling, matching, discrimination, and rating. However, many other types of emotion perception tasks exist that have not been studied so far but can be adapted to the fMRI environment (Boller & Grafman, 2000; Wilhelm et al., 2014). We thus invite future neuroimaging studies to test as many and as varied perception tasks as possible to reveal the neurocognitive processes behind them. Following our post hoc classifications of studies, labeling emotions was by far the most used emotion perception paradigm, whereas emotion rating and discrimination were the least used paradigms. Unfortunately, the number of experiments for emotion rating (*n* = 10) and emotion discrimination (*n* = 19) were below the minimum number generally recommended for neuroimaging meta‐analyses (Eickhoff et al., 2016). As such, the rate of false positives may be high, and results should be interpreted with caution. Furthermore, we were unable to properly compare the different stimulus modalities. The number of experiments per paradigm, emotion construct (e.g., joy, anger, fear), and modality (i.e., faces, voices, body postures) did not reach the minimum recommended number to draw meaningful conclusions (Eickhoff et al., 2016). Instead, we looked at the commonalities and distinctions between paradigms of emotion perception, while trying to mitigate the noise introduced by the different perceptual modalities and the various emotion constructs by applying a more stringent statistical analysis than typically employed by neuroimaging meta‐analyses, that is, a cluster‐forming threshold at voxel level of *p* < .0001 and a cluster‐level threshold of *p* < .01 (Eickhoff et al., 2012). We therefore encourage future empirical studies to simultaneously compare several emotion perception tasks on a wide range of stimuli (i.e., faces, voices, and body postures) in a full‐factorial design to reveal differences and commonalities between tasks and classes of stimuli.

## Data Availability

The data of the present meta‐analysis are available from the corresponding author upon reasonable request.
